# Retroperitoneal Abdominal Accessory Splenosis

**DOI:** 10.7759/cureus.15290

**Published:** 2021-05-28

**Authors:** Hatan Mortada, Hisham Alkhaldi, Awadh Alqahtani

**Affiliations:** 1 Department of Plastic Surgery and Burn Unit, King Saud Medical City, Riyadh, SAU; 2 Division of Plastic Surgery and Department of Surgery, King Saud University Medical City, Riyadh, SAU; 3 Department of Pathology, College of Medicine, King Saud University, Riyadh, SAU; 4 Department of Surgery, College of Medicine, King Saud University, Riyadh, SAU

**Keywords:** retroperitoneal mass, accessory spleen, histopathology, laparoscopy, abdominal pain

## Abstract

Accessory splenosis (AS) is an uncommon condition that is formed from the lateral dorsal mesogastrium due to the defective union of separate splenic masses. It is often found next to the pancreatic tail and the hilum part of the spleen. The retroperitoneal is described as an unusual and uncommon site. We present a case of a 39-year-old woman with an AS who came to the clinic with a right abdominopelvic mass. Computerized tomography (CT) scan revealed a right retroperitoneal abdominal mass. Diagnostic laparoscopy and resection of the retroperitoneal mass were done, and histological investigation showed that the removed mass was a spleen. Although retroperitoneal abdominal AS is an unusual condition, it should be considered in the list of abdominopelvic mass differential diagnoses.

## Introduction

Accessory splenosis (AS) is also known as splenules, supernumerary spleen, or splenunucli. AS embryologically develops during the sixth week after the spleen cells' deposition along the midline path, and more commonly occurs left-sided. The most common location of AS is the pancreas tail and spleen's hilum [1]. AS is considered a congenital structure due to the failure of union of the splenic primordium, originating from the dorsal mesogastrium's left side during the early phase of the fetus's life [2]. AS are found in about 10-30% of the autopsy findings [3] and are predominately asymptomatic; they are usually diagnosed incidentally on imaging or intra-operatively [4]. In this case study, we present an incidental finding of a retroperitoneal abdominal AS in a woman with unexplained abdominal pain whose final diagnosis was made after laparoscopic mass resection. Nonetheless, AS is unusual; the possibility of AS must be acknowledged in the retroperitoneal abdominal mass differential diagnosis.

## Case presentation

**Figure 1:**
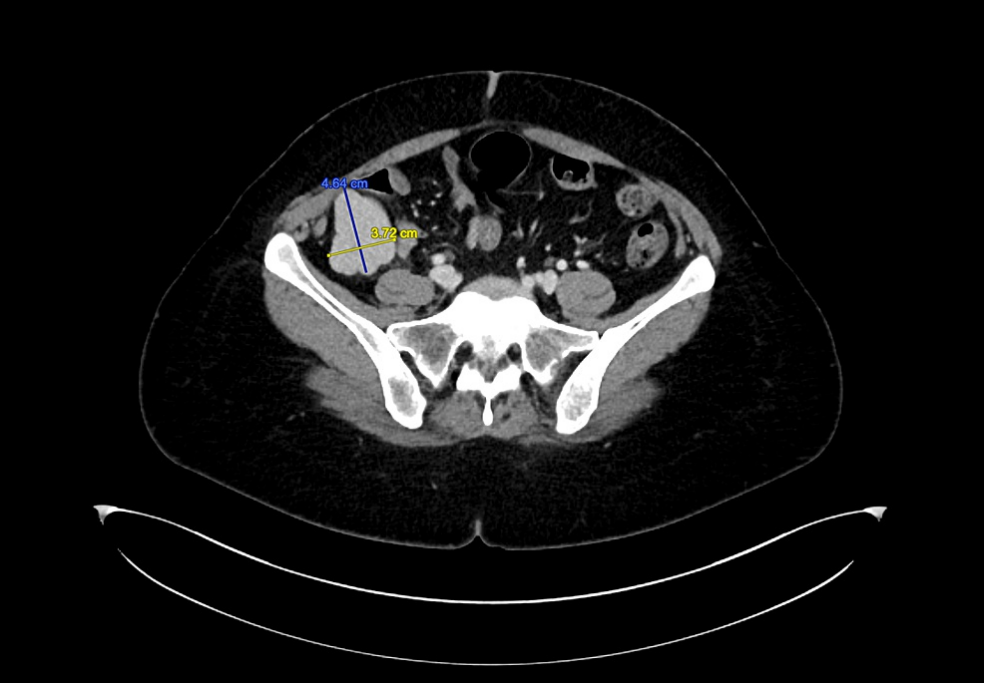
Axial CT scan of the abdomen and pelvis showing right lower quadrant enhancing mass with internal calcification, intense enhancement of the capsule due to paten vascular supply in the spleen’s capsule.

A 39-year-old lady was identified to have diabetes mellitus type 2, dyslipidemia, and gastroesophageal reflux disease. She complained of recurrent epigastric abdominal pain of five months duration. There was no nausea, vomiting, diarrhea, constipation, night sweating, weight loss, or history of trauma to the abdomen. The patient had no past surgical history. The results of the physical and abdominal examinations were unremarkable. Laboratory assessment, including inflammatory markers, were normal. Abdominopelvic computerized tomography (CT) scan showed a well-defined right lower quadrant lobulated soft tissue enhancing mass with internal calcification seen in relation to a short segment of terminal ileum (Figure 1).

**Figure 2:**
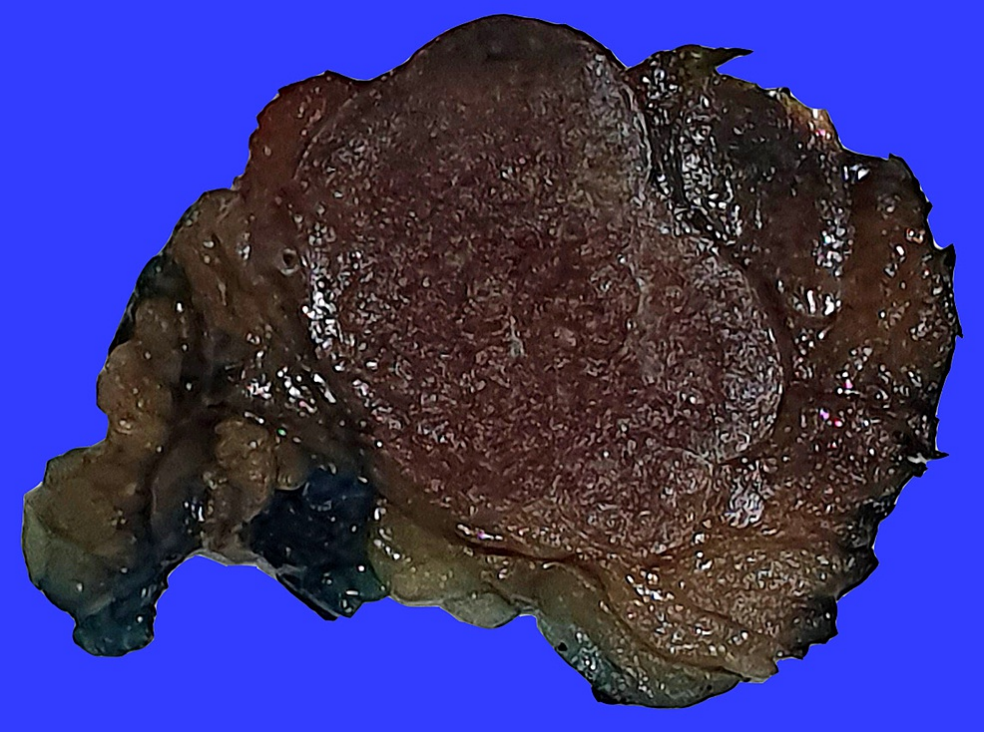
The gross examination of the mass cut surfaces shows a tan-brown nodular texture that characterize a spleen, and surrounding fat.

The small and large bowel loops were unremarkable with no signs of bowel obstruction. The appendix was seen filled with air in retrocecal and subhepatic locations with normal size. The liver showed no features of cirrhosis and no focal lesions. There was no biliary dilatation. The patient was referred to our service and underwent diagnostic laparoscopy and resection of retroperitoneal mass under general anesthesia; intraoperative findings include right lower quadrant mass lobulated with attachment to anterior/lateral abdominal wall. The appendix was normal. Later on, the resected mass was histopathologically confirmed as AS (splenule). It consisted of a grey, tan oval mass with a nodular outer surface and surrounding adipose tissue (Figure 2).

**Figure 3:**
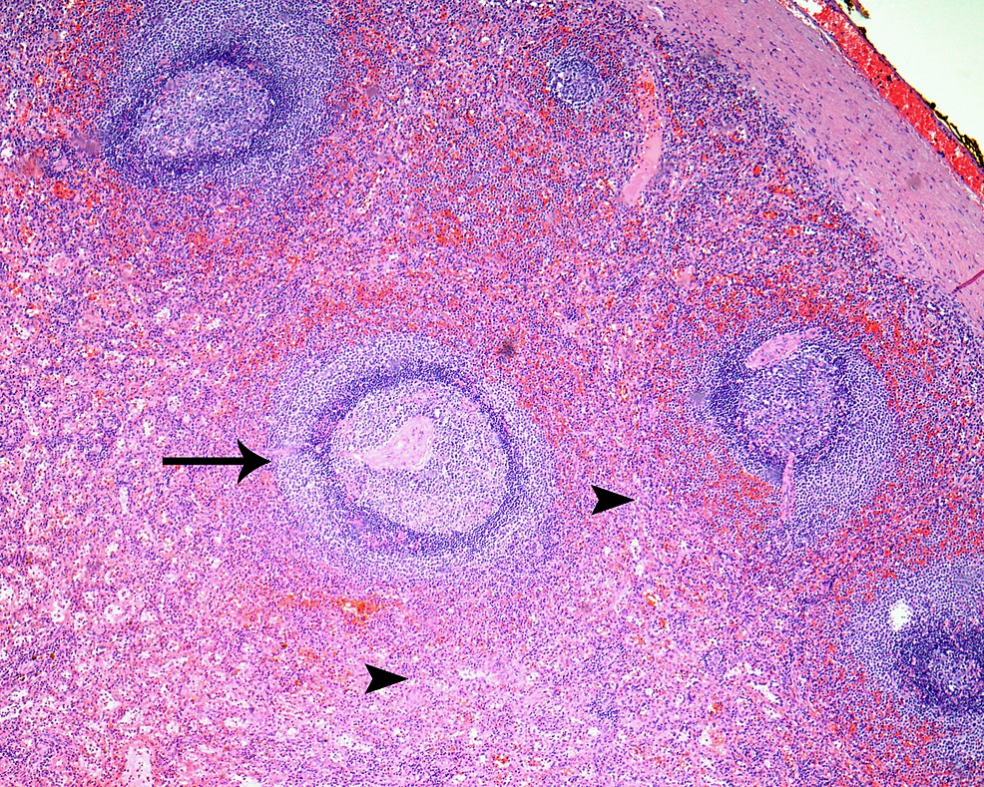
Microscopic examination confirmed the splenic tissue histology, which included white pulp (arrow), red pulp (arrow heads) and a capsule (upper right corner) (hematoxylin & eosin stain, 40x).

On gross examination, the mass measured 8 x 4 x 3 cm. Serial sectioning revealed heterogenous well-circumscribed red-tan and nodular cut surfaces with a focal area of calcification. Microscopic examination confirmed spleen histology. The sections showed the classic components of a spleen. The first is the splenic red pulp venous sinusoids and splenic cords. The second is the white pulp formed by sheaths of lymphoid cells around arteries with surrounding mantle zone and outer marginal zones (Figure 3). The postoperative period was uneventful, and the patient's complains subsided entirely after the operation.

## Discussion

Numerous embryological anomalies of the spleen have been discovered, including multiple spleens, complete agenesis, small extra accessory spleens, and polysplenia [5]. The localization of AS can vary broadly, but the most frequent sites are the spleen's hilum (75%), the tail of the pancreas (20%), and the greater omentum, at the level of the stomach's greater curvature [1,4,5]. Additionally, the serosa of the terminal ileum is an uncommon location. Thus, in this research case report, we presented an AS in one of the rarest locations. Acquired splenic tissue (splenosis) and congenital splenic tissue are the two types of ectopic splenic tissue (AS, splenunculi). Acquired ectopic splenic tissue (splenosis) is the auto-transplantation of spleen's tissue post-trauma or after splenectomy. In our case report, the patient had no previous history of splenectomy nor trauma; consequently, it is most likely that the AS was previously undiagnosed. Among the general population, congenital AS is estimated to be seen in 10-30%. A previous study by Unver Dogan et al. studied 720 autopsies, and found that 6.7% of the cases had AS, only two were retroperitoneal AS; therefore, the retroperitoneal is classified as a rare location [1]. Furthermore, the most common number of AS is on (85%), followed by two (14%), and very occasionally, three or more (1%), as seen in this case.

The exact size of the AS ranges from few millimeters to centimeters [1,5,6]. In females, AS occurs 13 times more often than in males, with an average age between 20 and 40 [7]. These are in agreement with the present case. A separate object is an abdominopelvic spleen, which results from the ligamentous splenic apparatus, which allows the spleen to move within the abdomen, which is not fully developed. Vural et al. have described a pelvic AS that was laparoscopically managed [8]. Azar et al. also described a middle-aged woman with pelvic AS, emphasizing the significance of including AS in pelvic masses differential diagnosis [7]. In most patients, AS is incidentally discovered and has no clinical significance, usually found during abdominal surgeries or radiological investigations [9], unlike the patient in this case report who presented with recurrent history of on and off abdominal pain for months. Therefore, AS should be considered in the differential diagnosis of retroperitoneal abdominal masses. Hence, our initial differential diagnosis was of a gastrointestinal stromal tumor, desmoid mass, or neuroendocrine tumor, including carcinoid.

## Conclusions

Despite retroperitoneal abdominal AS being uncommon in clinical practice, it is usually present as an asymptomatic retroperitoneal abdominal mass condition. A high index of suspicion should exist for any patient with AS, as well as it should be considered in the differential diagnosis of right lower quadrant soft tissue mass causing concurrent abdominal pain with enhancement in CT.
